# The Cone Optoretinogram as a Function of Retinal Eccentricity^†^

**DOI:** 10.3390/photonics12070676

**Published:** 2025-07-04

**Authors:** Raymond L. Warner, Peiluo Xu, David H. Brainard, Jessica I. W. Morgan

**Affiliations:** 1 Scheie Eye Institute, Department of Ophthalmology, University of Pennsylvania, Philadelphia, PA 19104, USA;; 2 Department of Bioengineering, University of Pennsylvania, Philadelphia, PA 19104, USA;; 3 Department of Psychology, University of Pennsylvania, Philadelphia, PA 19104, USA;; 4 Center for Advanced Retinal and Ocular Therapeutics, University of Pennsylvania, Philadelphia, PA 19104, USA

**Keywords:** adaptive optics, optoretinography, ophthalmic imaging

## Abstract

Adaptive optics scanning laser ophthalmoscopy optoretinography quantifies cellular function in the living retina by measuring the *en face* intensity change in cone photoreceptors due to visual stimulation. To fulfill the potential of optoretinography as a biomarker for assessing function in disease, we require normative optoretinographic measurements across the retina. Here we provide such measurements. We use a custom adaptive optics scanning laser ophthalmoscope to investigate cone optoretinogram (ORG) amplitudes across retinal eccentricity in five normal-sighted participants. For this purpose, we aggregated signals across cones in each measurement (~1° by 1° patch) to provide a measurement we call the population ORG. Average population ORG amplitudes decreased with increasing eccentricity for all participants, although there were individual differences in the detailed pattern of the decrease. ORG amplitudes were correlated with the thickness of the outer retina as measured using clinical OCT, which also decreases with eccentricity. Characterizing the population cone ORG as a function of eccentricity in normal-sighted participants is an important step towards establishing norms that will allow it to be used as a biomarker for assessing photoreceptor function in retinal disease.

## 1. Introduction

Retinal imaging has revolutionized ophthalmic medicine as clinicians and scientists can readily observe living tissue and use structural phenotypes to diagnose disease. Beyond standard clinical practice, state-of-the-art retinal imaging techniques enable high-resolution visualization of individual cells within the eye. For example, adaptive optics (AO) ophthalmoscopy, which measures and corrects for the eye’s optical aberrations, allows visualization of the individual photoreceptors within the photoreceptor mosaic. Indeed, over the past three decades photoreceptor mosaic structure has been investigated in several disease conditions [1–5].

Until recently, the spatial resolution available for measuring photoreceptor function has lagged that of structural assessments. The emerging field of optoretinography, however, is now enabling objective measurement of photoreceptor function at a fine spatial scale [6]. In contrast to the well-known electroretinogram, which measures an electrical signal in response to visual stimulation [7], the optoretinogram (ORG) measures an optical response to stimuli [8] using both *en face* and cross-sectional imaging modalities [9–23]. Recent studies have shown that the ORG increases with stimulus irradiance [18,20], varies predictably with stimulus wavelength [11,19,20], and is repeatable [9]. Studies have also found an attenuated ORG in cases of choroideremia [24] and retinitis pigmentosa [25–28].

Much remains to be learned about the ORG, including how it varies with photoreceptor morphology in the normal and diseased retina. Normal cone structure varies with retinal eccentricity, with foveal cones being the thinnest, longest, and most densely packed [29]. Cone aperture increases with eccentricity [29], while cone outer segment length decreases [30]. In addition, cone density decreases with retinal eccentricity [31]. Most recently, studies have begun characterizing the relationship between retinal eccentricity and ORG with differing methodologies and imaging modalities [12,25–27,32], as one step towards translating the ORG to studies of disease. Here, we add to this literature by reporting ORG amplitudes measured from *en face* cone photoreceptor images at different retinal eccentricities acquired using AO scanning laser ophthalmoscopy. Specifically, we investigate the ORG for a population of cones within ~1° by 1° imaging regions at five retinal locations, in five normal-sighted participants.

## 2. Materials and Methods

### 2.1. Participants

This research adhered to the tenets of the Declaration of Helsinki and was approved by the Institutional Review Board at the University of Pennsylvania. Five participants with no known retinal pathologies (ages 22–43 years) volunteered for the study. Each participant’s best-corrected visual acuity was confirmed to be at least 20/20.

### 2.2. AOSLO System

A schematic of the optical design of the adaptive optics scanning laser ophthalmoscope (AOSLO) system has been previously described in Figure 5 of Dubra and Sulai., 2011 [33] with minor modifications for the ORG imaging application presented here. Briefly, the custom AOSLO used an 840 Δ26 nm super-luminescent diode as the wavefront sensing source (Superlum, Cork, Ireland) and a deformable mirror with 97 actuators to correct for both lower-order and higher-order aberrations (Alpao SAS, Montbonnot-Saint-Martin, France). Aberrations of the eye at each actuator were measured with a Shack–Hartmann Wavefront Sensor and controlled in a closed-loop configuration using the deformable mirror at a frame rate of 8.2 Hz. For structural imaging sessions, the system utilized a 795 nm Δ15 nm super-luminescent diode (Superlum, Cork, Ireland) as the imaging source. For our ORG application, the AOSLO system described in detail in [33] was modified as follows. First, the super-luminescent diode was replaced with a 785 nm luminescent diode (Thorlabs, Newton, NJ, USA; LB785-SF20, 95 μm coherence length) as the imaging source. This modification increases the coherence length of the imaging source, and we have found that we obtain a larger ORG signal with the increased coherence length. Second, a super-continuum laser (NKT Photonics, Birkerød, Denmark; SuperK EXTREME/VARIA) was added, with its light merged with the optical path of the imaging beam as the visible-light stimulus for ORG imaging. Third, to control the exposure of the 545 Δ10 nm visible stimulus, a mechanical shutter (Thorlabs, SHB05T) was positioned in front of the visible light fiber and synced with the slow scan to open and close at designated times relative to the AOSLO frame acquisitions. All three light beams were coaxially aligned and scanned at a temporal frequency of 17.85 frames per second to generate a ~1° × 1° raster on the retina. Average irradiance at the retina was 100 μW/deg^2^ for the 785 nm luminescent diode, 10 μW/deg^2^ for the 840 nm superluminiscent diode, and 770 nW/deg^2^ for the 545 Δ10 nm visible stimulus. Light reflected from the retina at 785 nm was collected using three photomultiplier tubes: one to collect directly back-scattered light in a confocal modality, and two to collect multiply-scattered light in a split detection configuration for visualization of cone outer segment morphology [34].

**Figure 1.**
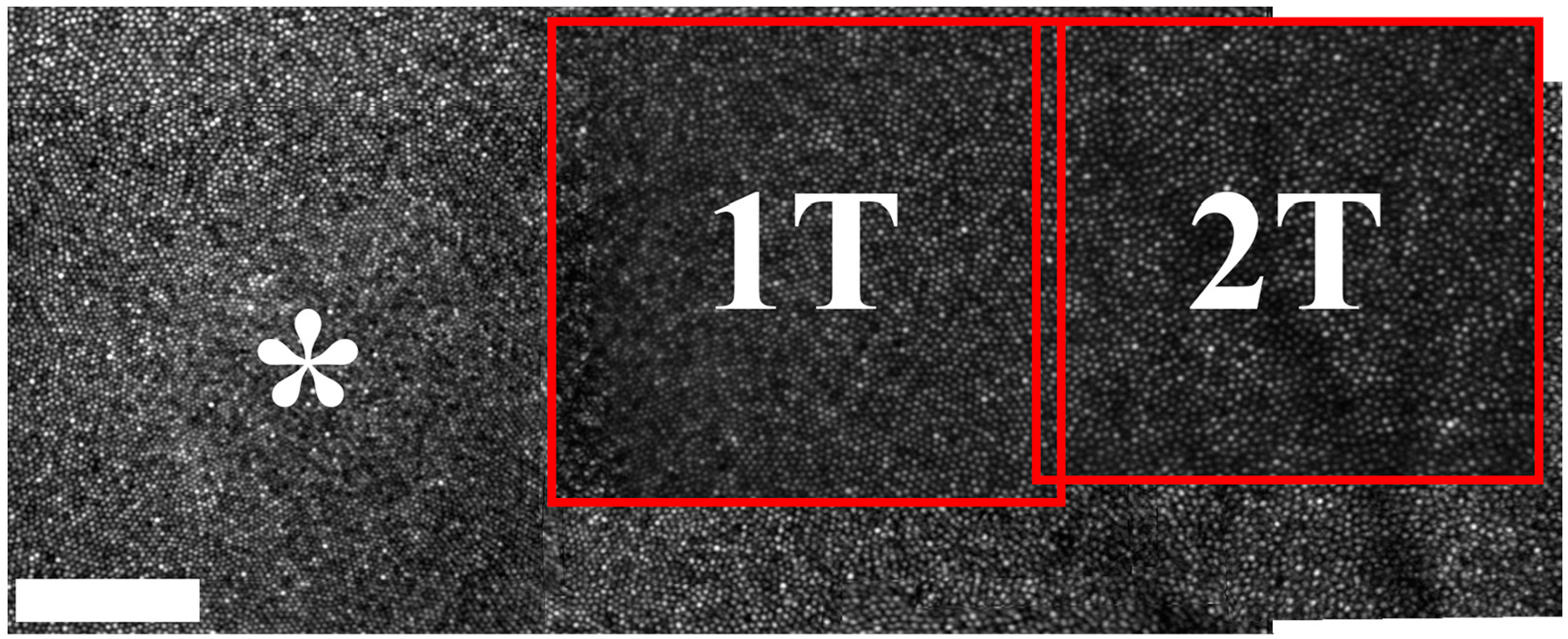
A zoomed-in montage of the photoreceptor mosaic of participant 11108 with two of the five ROI locations shown. The full montage of the fovea and temporal retina of participant 11108 is provided as Supplementary Figure S1. Asterisk indicates participant’s fovea. Scale bar is 100 μm.

Prior to ORG imaging, structural images of the cone mosaic surrounding the fovea and along the horizontal and vertical meridians were acquired in a separate session to ensure sufficient participant compliance and acquisition of high-quality images, using the AOSLO described above. Images were registered and montaged across all four meridians [35] as previously described (Figure 1; Supplementary Figure S1) and five retinal locations along the temporal meridian (denoted 1T, 2T, 4T, 8T, and 16T with the number indicating eccentricity in degrees) were used for ORG measurements in the functional imaging session.

### 2.3. AOSLO Optoretinography Imaging Protocol

At the beginning of each imaging session, the participant’s preferred eye was dilated with one drop of 2.5% phenylephrine hydrochloride ophthalmic solution and one drop of 1% tropicamide ophthalmic solution. A “video” in the dataset is a 93-frame AOSLO image sequence. A “set” in this study refers to the collection of six consecutive videos at the same retinal location where the first three consecutive videos were collected with no stimulus presentation (“control condition”) and the second three consecutive videos were collected with a 168 ms (three video frames) visible stimulus (“stimulus condition”). Prior to collecting a set at one retinal location, participants were dark adapted for three minutes. Between video recordings within a set, participants were asked to blink several times to refresh their tear film. Once the six consecutive videos of the set were collected, the participants again dark adapted for 3 min, and the procedure was repeated at the next retinal location. Once all five locations had been imaged, the process was repeated, such that the complete dataset included 5 sets at each location (15 control and 15 stimulus videos at each location). The order in which retinal locations were tested was randomized for each set for each participant.

### 2.4. Image Registration

To quantify the change in cell intensity over time, videos were processed via strip-alignment registration [35]. First, frame distortions resulting from sinusoidal horizontal scanning were corrected. Then, multiple reference frames within one second of the stimulus onset were automatically selected based on features such as brightness, contrast, and over-lap with other frames in the video, and the video was registered. Across these candidate reference frames, the one that led to the registered video with the most successfully registered frames was then chosen as the registered video that continued through the analysis pipeline. Registered versions of the video generated using the other candidate reference frames were not analyzed further. If fewer than 60% of the frames within one-second of the stimulus onset were unable to be registered in the video selected for analysis, the video was excluded from further analysis. In addition, an image for the registered video selected for further analysis was generated by averaging all the registered frames within the registered video (“all frames” image).

The “all frames” images from each video at each retinal eccentricity were co-registered to the “all frames” image with the largest area (across videos) using constellation matching [36]. A location-specific image (“all videos” image) was generated by averaging the co-registered images. The image transformations for each co-registered “all frames” image was then applied to each registered video such that all frames from all videos in a specific location were aligned to the “all videos” image. This was accomplished for both confocal and split-detection modalities. Cone photoreceptors were identified semi-automatically at 1T and 2T [37] and manually at 4T, 8T, and 16T using MOSAIC (Translational Imaging Innovations) on the “all videos” image. Rod photoreceptors were excluded from further analysis (Supplementary Figure S2). Cones for each video were also semi-automatically identified using the “all frames” images for each video, and cone locations for each video were matched to cone locations from the “all videos” image. A binary mask was created from a motion contrast algorithm across all acquisitions, and cones in underlying vasculature features identified based on the motion contrast were excluded from analysis [38].

### 2.5. Intensity ORG Extraction

**Figure 2.**
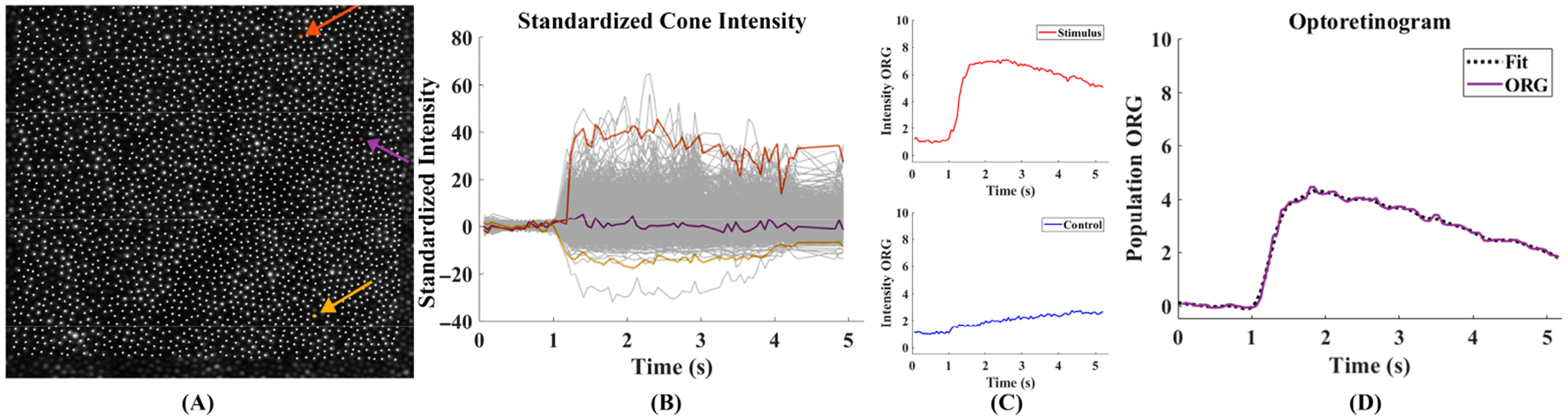
(**A**) An illustration of the identified cones from an individual “all frames” image for one retinal location for one participant. (**B**) Each cone’s intensity signal was standardized based on the cone’s pre-stimulus intensity. The three highlighted cone traces illustrate the heterogeneity in intensity across a population of cones. (**C**) The population response from the aggregated control (blue line) and stimulus (red line) videos. (**D**) The population ORG (purple line) is the difference between the stimulus and control population responses. A smoothing spline is fit to the ORG.

The population cone intensity ORG was measured using a methodology established previously [20], with the one adjustment that we increased the number of pixels over which we averaged to obtain the response for each cone as eccentricity increased. This was done to account for the increasing size of the cone apertures with eccentricity. In this study, a 3 × 3 pixel region for each cone at 1T and 2T, a 9 × 9 pixel region for each cone at 4T, and a 15 × 15 pixel region for each cone at 8T and 16T were averaged throughout the aligned videos. Each cone intensity signal throughout the video was divided by the mean intensity of all cones in the same frame. We standardized an individual cone’s intensity signal to its pre-stimulus mean and standard deviation (Figure 2B). To measure a video’s population cone response at each video frame, we took the standard deviation of the standardized cone responses in each frame. This was done separately for the stimulus and control videos. We then combined the standard deviation traces from multiple stimulus and control videos using the pooled standard deviation, defined as the square root of the mean squared standard deviation traces (Figure 2C). The population ORG was then calculated as the difference between the stimulus and control responses (Figure 2D). Lastly, a smoothing spline was fit to the ORG time course and the peak of the fit was taken as the ORG amplitude (MATLAB MathWorks, Natick, MA, USA, R2020b, function fit: “smoothing spline” method, smoothing parameter 0.9995).

### 2.6. Measurement of Outer Retinal Thickness

A B-scan through the fovea and along the horizontal meridian for each participant was acquired using a clinical Optical Coherence Tomography (OCT) device (Heidelberg Spectralis, Heidelberg, Germany) during a separate, independent session. The distance between the Ellipsoid Zone and the Retinal Pigment Epithelium/Bruch’s Membrane (EZ-to-RPE/BrM Distance) was estimated for each participant at each imaged location as previously described [39]. In brief, to estimate the EZ-to-RPE/BrM distance, we quantified the reflectivity as a function of depth centered at each of the retinal locations where the ORG was measured. Reflectivity was averaged over a lateral region (approximately 300 μm) at each depth. The locations of peak reflectance for the EZ and RPE/BrM layers were then extracted, and the distance between these two locations was calculated at each retinal eccentricity for each study participant (MATLAB^™^ MathWorks, Natick, MA, USA, R2020b).

### 2.7. Statistical Analysis

Statistical analysis was implemented using MATLAB^™^ (MathWorks, Natick, MA, USA, R2020b). A two-way ANOVA was used to compare ORG amplitudes between participants and retinal eccentricities with a significance level set at *p* < 0.05. Linear regression was used to characterize the relation between ORG amplitude and EZ-to-RPE/BrM distance.

### 2.8. Pre-Registration Documentation

This study was pre-registered at the Open Science Foundation (https://osf.io/n759w/, uploaded 19 August 2023) prior to initiation of data collection. We adhered to the imaging protocol and analysis guidelines as written, although we do not present here any single cone analyses. The EZ-to-RPE/BrM distance analysis was not described in the pre-registration document and was developed after data collection.

## 3. Results

### 3.1. Intensity ORG Decreases Across Retinal Eccentricity

**Figure 3.**
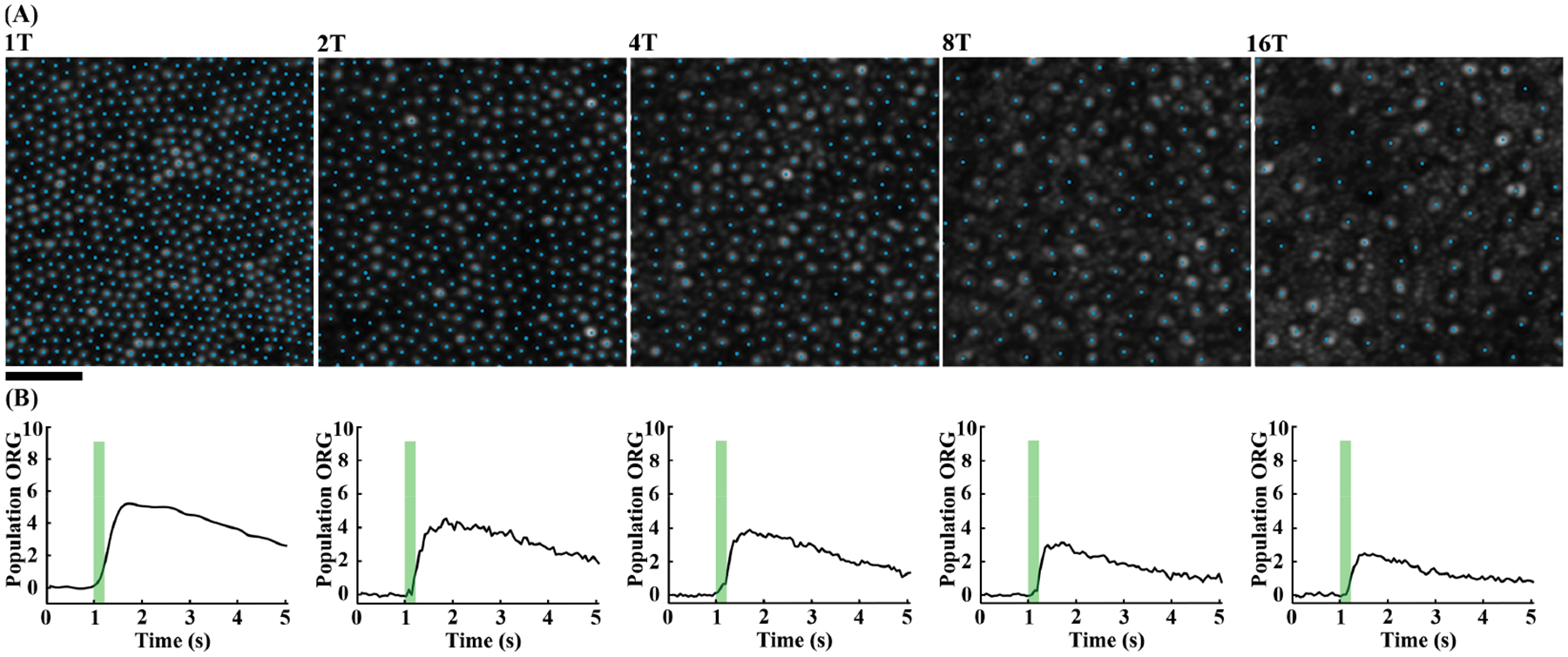
(**A**) AOSLO images for participant 11108 at 1T, 2T, 4T, 8T, and 16T temporal retina with cone locations identified. Images are cropped for visualization of identified cones. Scale bar = 25 μm. (**B**) The corresponding population ORG at each retinal location. Vertical green bars indicate the onset and duration the 545 nm visible light stimulus.

Cone mosaics were successfully imaged, and the population cone ORG was successfully extracted for all locations in all study participants. Figure 3 shows cropped “all videos” images (Figure 3A) and population cone ORG measurements (Figure 3B) at each location for one participant. The ORGs vary similarly with time at all locations, with a rapid rise in response followed by a slow decay. As eccentricity increases, there is a systematic decrease in the amplitude of the ORG response.

**Figure 4.**
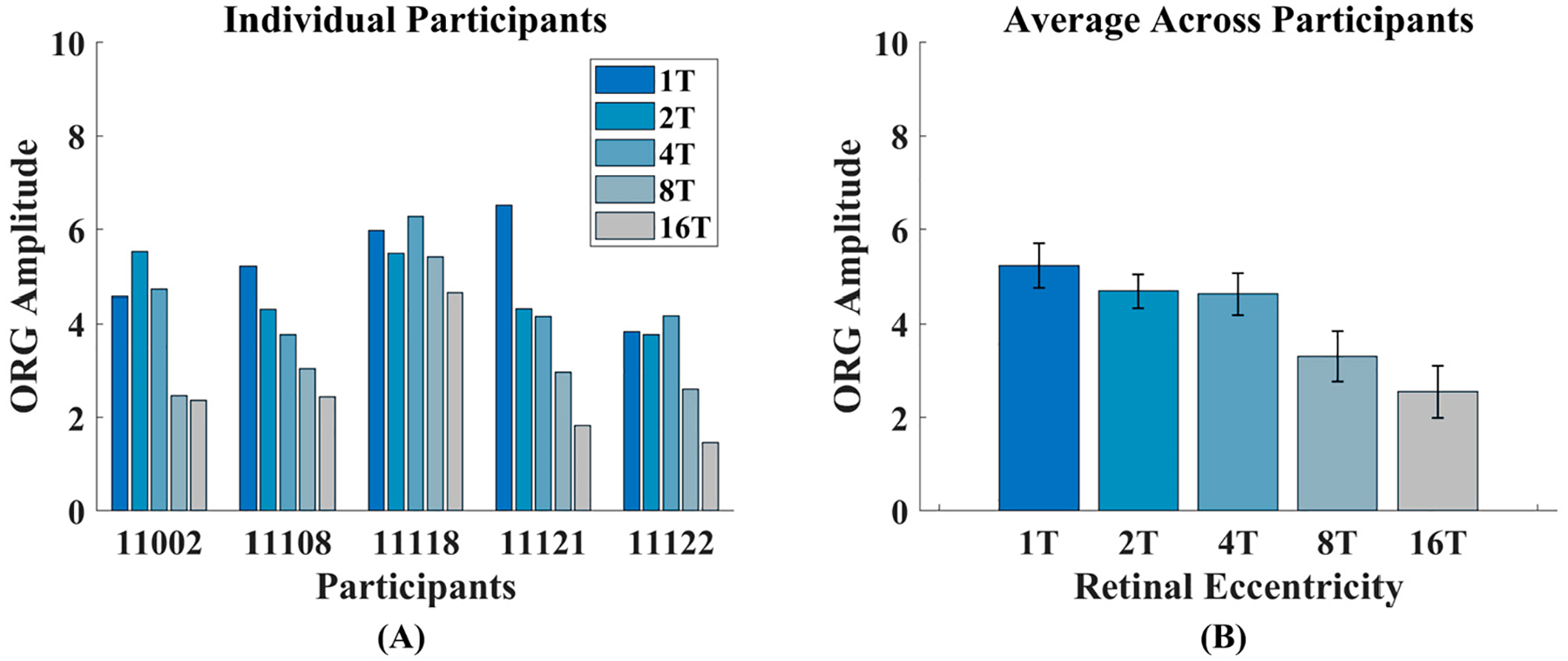
(**A**) The ORG amplitude for each participant at each retinal location. (**B**) The ORG amplitude averaged across all participants at each retinal location. Error bars represent standard error of the mean.

To visualize this more clearly, Figure 4A shows the ORG amplitudes across retinal locations for all participants. ORG amplitudes decrease with increasing eccentricity for all participants: all participants exhibit lower ORG amplitudes at 8T and 16T relative to 1T, 2T, and 4T. Some variability exists between participants across the more foveal eccentricities. On average however, the ORG decreased with eccentricity, with the average ORG amplitude at 16T being approximately half that at 1T (Figure 4B). ORG amplitudes were 5.22 a.u. ± 0.48 SEM at 1T, 4.68 a.u. ± 0.35 SEM at 2T, 4.62 a.u. ± 0.35 SEM at 4T, 3.30 a.u. ± 0.35 SEM at 8T, and 2.55 a.u. ± 0.35 SEM at 16T. A two-way ANOVA showed a significant main effect of eccentricity [F (4,24) = 8.97, *p* < 0.001] and participants [F (4,24) = 13.88, *p* < 0.001]. Each participant’s individual ORG amplitudes are provided in Supplementary Table S1.

### 3.2. Increased Outer Retinal Thickness Correlates with ORG Amplitude

**Figure 5.**
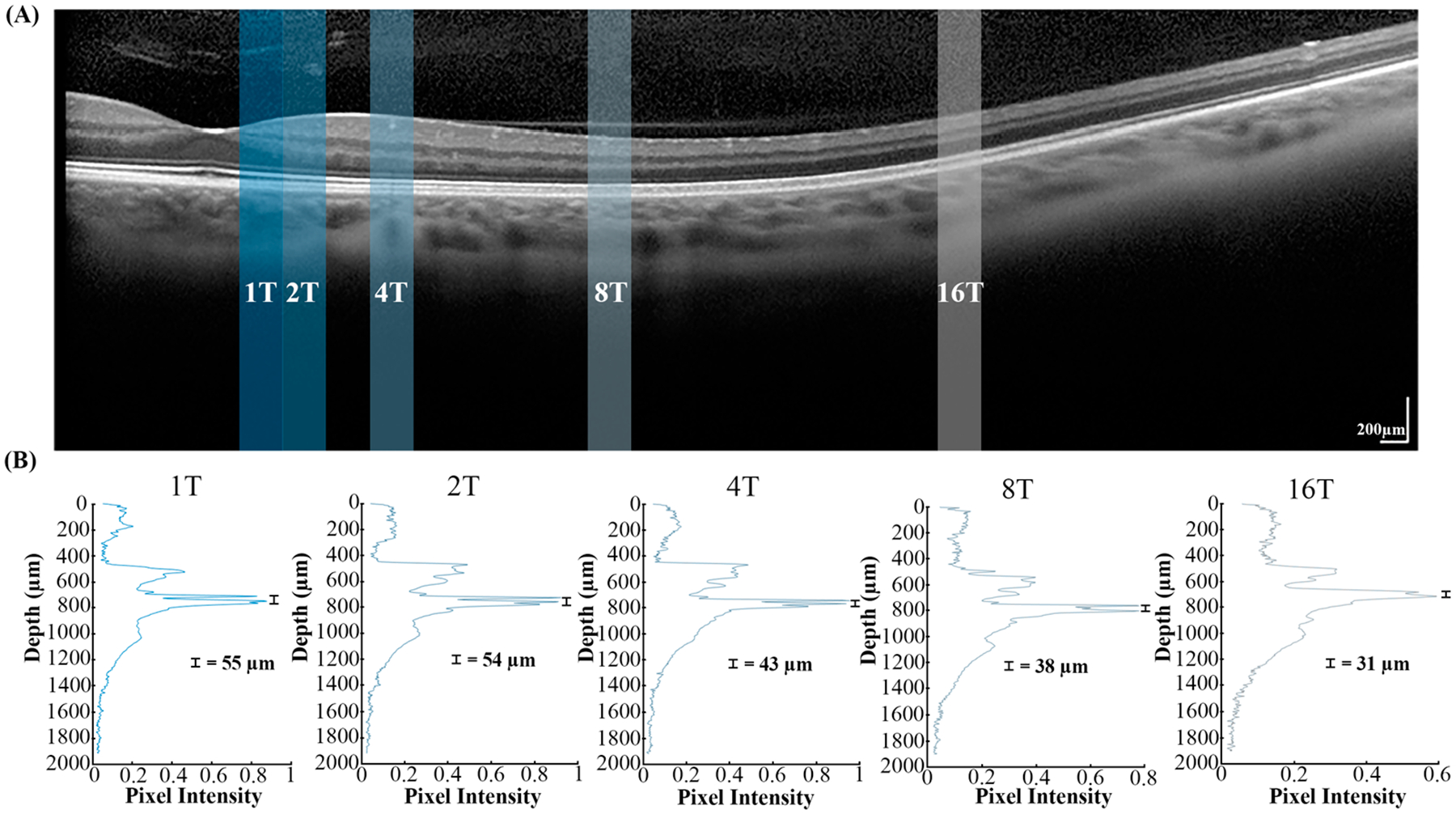
(**A**) OCT B-scan (Heidelberg Spectralis) of the temporal retina of participant 11002 and (**B**) line reflectivity profiles at each ROI. The colored vertical bars overlaying the image show the region where pixel intensities were averaged horizontally (over a 300 μm width) at each retinal eccentricity to obtain the line reflectivity profiles. The vertical black bars on the line reflectivity plots show the measured distance between the EZ and RPE/BrM.

**Figure 6.**
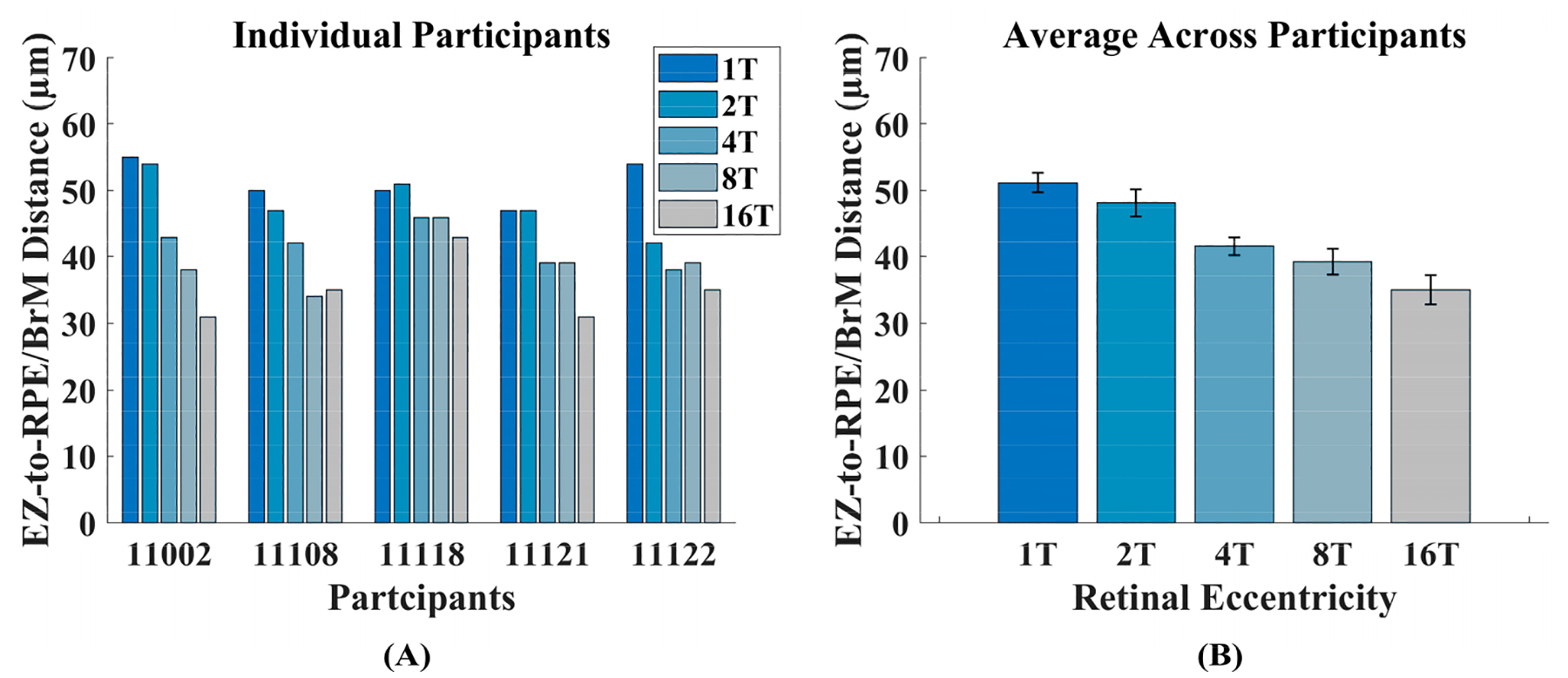
(**A**) The EZ-to-RPE/BrM distance for each participant at each ROI. (**B**) The EZ-to-RPE/BrM distance averaged across participants at each retinal eccentricity. Error bars represent standard error of the mean.

In all the participants, outer retinal thickness (taken as EZ-to-RPE/BrM distance) decreased with increasing retinal eccentricity, although not monotonically in all cases. Figure 5 highlights both how we estimated EZ-to-RPE/BrM distance as well as the decrease in this distance with increasing eccentricity for one participant. This relationship is shown for each participant in Figure 6A. When averaged, Figure 6B shows a decreasing trend across retinal eccentricity (51.2 μm ± 0.89 SEM at 1T, 48.2 μm ± 1.46 SEM at 2T, 41.6 μm ± 0.92 SEM at 4T, 39.2 μm ± 0.33 SEM at 8T, 35 μm ± 0.45 SEM at 16T). A two-way ANOVA showed only a significant main effect of eccentricity [F (4,24) = 18.02, *p* < 0.001] and not of participants [F (4,24) = 2.97, *p* > 0.05]. Supplementary Table S1 contains EZ-to-RPE/BrM distance measurements at each ROI for each participant.

**Figure 7.**
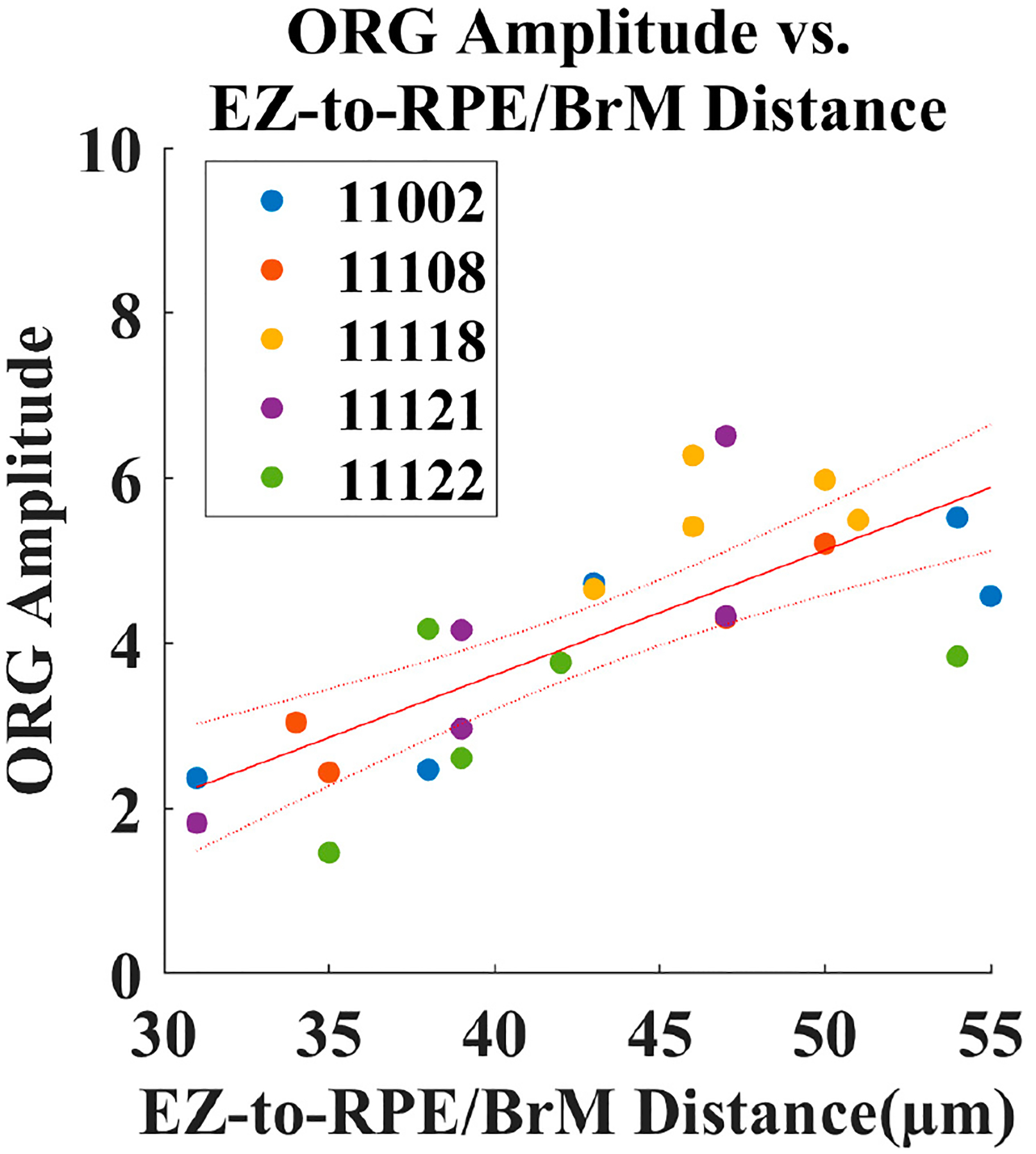
The ORG amplitude for each participant at each ROI plotted against EZ-to-RPE/BrM distance. The data was fit by linear regression (solid red line) with corresponding 95% confidence intervals (dotted red lines). The fit shows a significant linear relation between ORG amplitude and EZ-to-RPE/BrM distance, with ORG amplitude increasing with EZ-to-RPE/BrM distance (*p* < 0.001).

Figure 7 shows the relationship between ORG amplitude and EZ-to-RPE/BrM distance. Each point represents data from one location for one subject. The x-axis shows the EZ-to-RPE/BrM distance and the y-axis shows ORG amplitude. This representation allows visualization of the relationship in a manner that leverages both variation across subjects and eccentricity. A linear fit indicates that ORG amplitude increases systematically with EZ-to-RPE/BrM distance, with a correlation coefficient of R^2^ = 0.58.

## 4. Discussion

With AOSLO imaging, measuring cellular changes in the human retina provides a tool for characterizing cone photoreceptor function. In this study, we have shown that the population cone ORG amplitude decreases with retinal eccentricity in normal-sighted participants (Figure 4). Additionally, we have also shown that this decrease in ORG amplitude correlates with EZ-to-RPE/BrM distance (Figure 7).

Our investigation of the ORG in normal-sighted participants complements other studies investigating the cone ORG. Previous studies have examined ORG response as a function of stimulus intensity [18,20] and wavelength [11,17,20]. Furthermore, when investigating the ORG in normal-sighted participants, it has proven to be a repeatable and reliable measurement both within and across sessions [9]. This study, in correspondence with other literature investigating the ORG response, aims to establish the ORG as an objective biomarker of photoreceptor function in the clinical population. By characterizing the ORG in normal-sighted participants across various facets of methodologies and imaging modalities, future studies can be developed to assess photoreceptor function in the presence of retinal disease.

Investigating the relationship between ORG amplitude and retinal eccentricity is an ongoing project for the field, in both normal [12,25] and diseased retina [25,26]. We have shown that the average ORG amplitude decreases with increasing retinal eccentricity. This relationship has also been exhibited in Gaffney et al., 2024 [25] using similar methodologies including using an AOSLO to capture the intensity ORG of *en face* cone intensity changes. Jiang et al., 2022 [12] and Wendel et al., 2024 [27] likewise find a decrease in ORG amplitude with increasing eccentricity using both non-AO and AO-OCT devices. In those studies, ORG is quantified as a change in the optical path length of the cone outer segment. Despite the different imaging and quantification techniques, the results across the multiple studies largely agree. For example, when averaged across participants, we found a 10% decrease in ORG at 2T in comparison to 1T. This is similar to the range shown in the Gaffney, Jiang, and Wendell studies where ORG amplitudes at 2T were reduced 8–13% in comparison to 1T [12,25,27]. While more variability is seen at the 4T locations between the studies, our findings fall within the reported ranges in comparison to 1T. To our knowledge, no other studies extended their measurements to 16T. Our current finding that ORG amplitude continues to decrease with eccentricity past 8T shows additional eccentricities, and retinal locations will need to be studied to fully characterize the normative properties of the ORG. Understanding the normative ORG at larger eccentricities is especially important for diseases such as retinitis pigmentosa and choroideremia where degeneration begins at peripheral locations and progresses towards the fovea [40,41].

It is believed that the ORG arises from a change in the outer segment optical path length [42]. When capturing an *en face* video recording of a cone photoreceptor, the intensity of the photoreceptor in the image depends in part on the interference between light reflected from the inner segment/outer segment junction and the cone outer segment tip [43]. A change in the optical path length between these surfaces will cause a change in the interference and thereby a change in the observed intensity of the cone in the image [10,15]. With this mechanism in mind, it is therefore reasonable to consider whether outer segment length correlates with the ORG. Here, we found that ORG amplitude was correlated with EZ-to-RPE/BrM distance (Figure 7), with that distance serving as a proxy for outer segment length. Similarly, other studies have also found that the ORG correlates with outer segment length [12,25,27]. In the present study, we choose to evaluate the EZ-to-RPE/BrM distance, as the interdigitation zone (also termed the cone outer segment tip) band is not always visible in a clinical grade OCT (Figure 4, 16T location). This is a limitation of the present study. Other techniques with greater axial resolution, in particular AO-OCT, can quantify the cone outer segment length at a cellular level [11,27]. Indeed, our measurements of EZ-to-RPE/BrM distance are larger than other reported measurements of outer segment length [12,25,26]. This is unsurprising as EZ-to-RPE/BrM distance includes both the outer segment and the RPE cell thicknesses. EZ-to-RPE/BrM distance, however, has been demonstrated to be useful for understanding outer segment health in cases of retinal disease [39] and therefore may be useful in translational studies where the interdigitation zone may not be visible.

**Figure 8.**
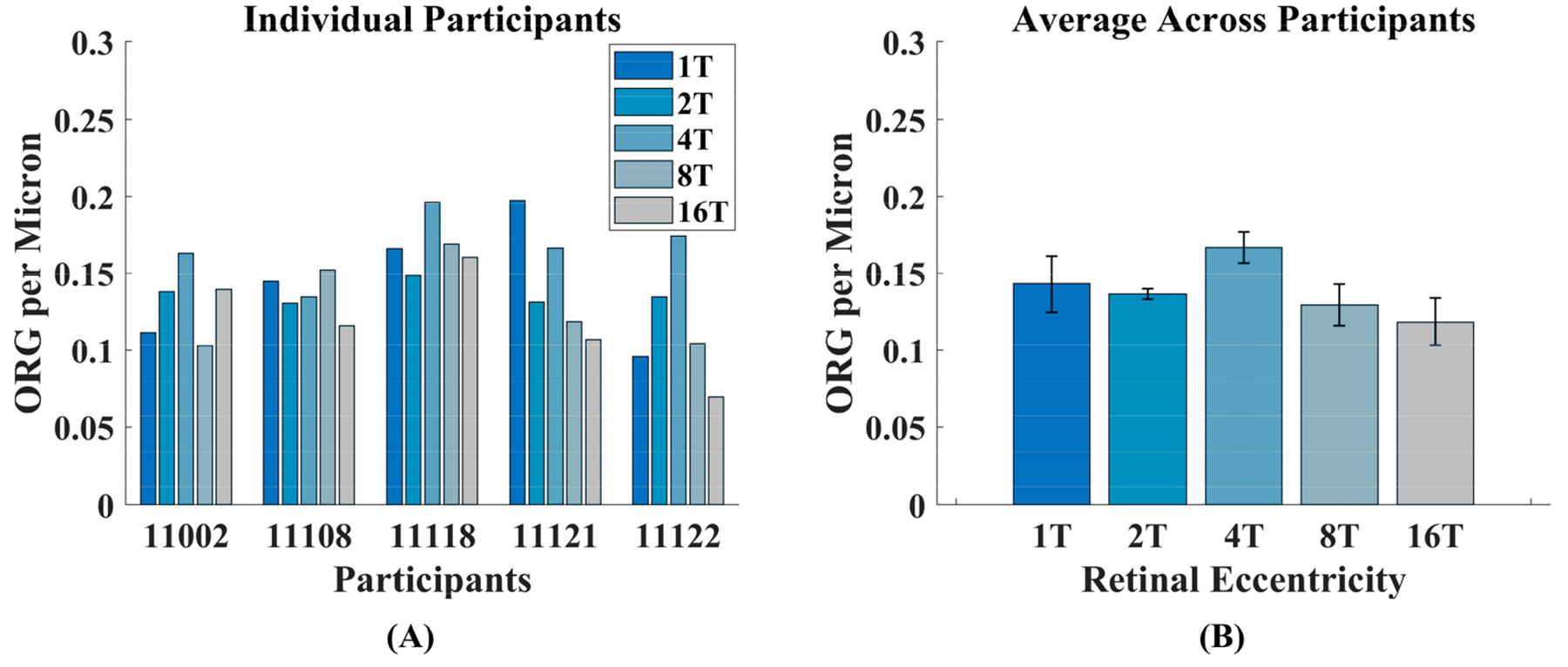
(**A**) The ORG amplitude per micron of outer segment for each participant at each retinal location. (**B**) The ORG amplitude per micron of outer segment averaged across all participants at each retinal location. ORG per micron is calculated as ORG amplitude/(EZ-to-RPE/BrM Distance—14 μm), where the RPE cell height is taken as 14 μm at all locations. Error bars represent standard error of the mean.

To extend the result shown in Figure 7, we estimated outer segment length by assuming an RPE cell height of 14 μm [44] and subtracting this distance from the measured EZ-to-RPE/BrM distances. We then divided the measured ORG amplitudes by the estimated outer segment lengths for each participant at each eccentricity. Figure 8 shows the ORG amplitude per μm of outer segment for each location. As with the ORG amplitudes, the ORG amplitude per μm of outer segment varies between participants, but there is no obvious trend over eccentricity (see average data in Figure 8B). A two-way ANOVA showed a significant main effect of participant [F (4,24) = 3.08, *p* < 0.05] but not eccentricity [F (4,24) = 2.70, *p* > 0.05]. This supports the idea that ORG amplitude depends, at least in part, on outer segment length.

Beyond outer segment length, other metrics of the cone mosaic and cone morphology vary with eccentricity. For example, cone density decreases while cone aperture size increases with eccentricity [29,45–52]. We do not believe our ORG measurements were affected by the different cone densities at different eccentricities or the number of cones included in the analysis (Supplemental Table S1), as our previous work established repeatable ORG amplitudes with different numbers of cones included in the analysis [20,24]. Future work will be required to determine whether the observed differences in ORG with eccentricity are mediated exclusively by changes in outer segment length, or whether cone aperture size, cone volume, or some other variables also play a role. While speckle may be present in individual images, its influence is minimized for cone identifications by selecting cones in the “all videos” image, which is comprised of many frames averaged together (Supplemental Figure S2), and minimized in the ORG measurements by averaging intensities from several pixels over the cone aperture and subtracting control from stimulated signals.

In addition to an effect of eccentricity on ORG amplitude, we also found a significant main effect of participant, showing that inter-individual differences contribute to the variability observed in ORG amplitudes. It has been suggested that inter-individual differences in axial length and outer segment length may contribute to the inter-individual differences observed in ORG, as these differences may alter the photons per unit area of the stimulus on the retina and the number of photopigment isomerizations that occur in response to a given stimulus [12]. Future studies will be needed to determine what effect other inter-individual differences, including biological variables such as age, sex, and race, have on the ORG.

Understanding how the ORG varies in the healthy retina is a prerequisite for using the ORG as a biomarker in disease. Recently, studies examining the ORG in the diseased retina have shown a decrease in ORG response relative to healthy retina [24–27]. These studies have also shown changes in photoreceptor morphology [25–27] and correlation between reduced ORGs and reduced retinal sensitivities [24,27]. The high potential for the ORG to be used as a measure of disease severity and an endpoint to assess therapeutic response gives further motivation for studies that fully characterize the normative ORG range.

## 5. Conclusions

We have shown that the population cone ORG decreases across retinal eccentricity in the normative population. Measuring the ORG across retinal eccentricities in normal-sighted participants is a significant step towards establishing normative ORG measures for future applications to assess cone function in the healthy and diseased eye.

## Supplementary Material

Supplemental File

The following supporting information can be downloaded at: https://www.mdpi.com/article/10.3390/photonics12070676/s1, Table S1: Measurements Across Individual Participants. Figure S1: Example photoreceptor montage and regions of interest. Figure S2: Example cone identifications.

## Data Availability

The data supporting the conclusion of this manuscript will be made available by the author.
