# Supplementary material for: The Cone Optoretinogram as a Function of Retinal Eccentricity
Source: Photonics. Author manuscript; Available in PMC 2026 Mar 13. (PMC12981616; doi:10.3390/photonics12070676)
Supplement: Supplemental File [file NIHMS2097118-supplement-Supplemental_File.docx]

**
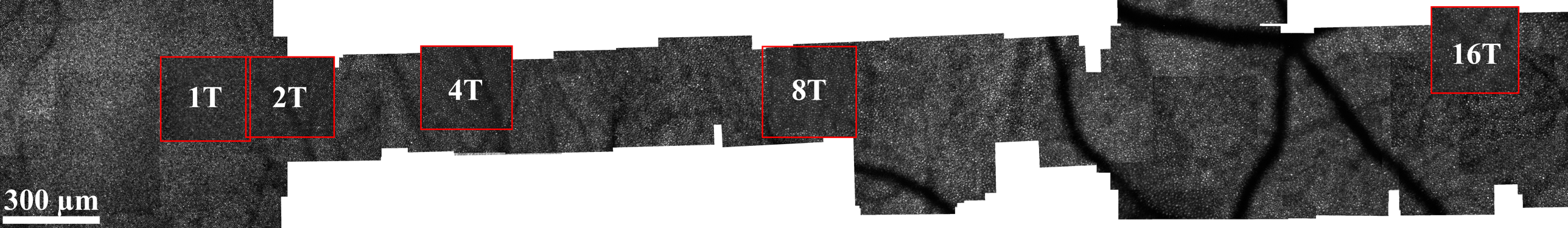
**

**Supplementary Figure 1.** A montage of the photoreceptor mosaic of participant 11108 showing the five ROI locations tested with ORG.


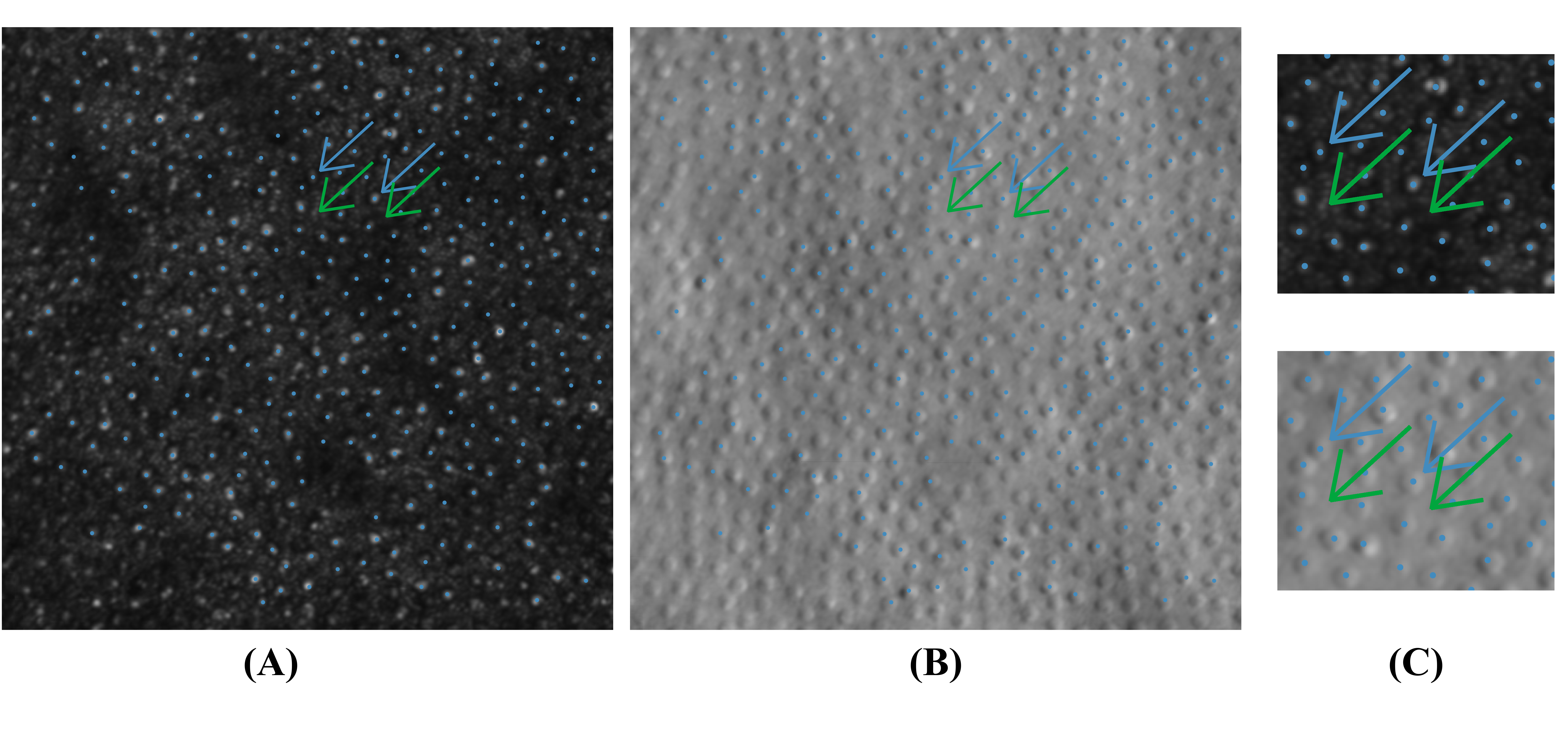


**Supplementary Figure 2.** The photoreceptor mosaic at 16T in participant 11108 in the (A) confocal and (B) split-detection modality and (C) a cropped area of both confocal and split-detection. Blue arrows highlight the cones in both modalities, while the green arrows illustrate rods. Blue dots represent the cone locations used for the ORG analysis while rod locations are excluded from further analysis.

|  | 1T | | | 2T | | | 4T | | | 8T | | | 16T | | |
| --- | --- | --- | --- | --- | --- | --- | --- | --- | --- | --- | --- | --- | --- | --- | --- |
|  | Cones | Amplitude (a.u) | EZ-to-BrM  Distance (µm) | Cones | Amplitude (a.u) | EZ-to-BrM  Distance (µm) | Cones | Amplitude (a.u) | EZ-to-BrM  Distance (µm) | Amplitude (a.u) | Amplitude (a.u) | EZ-to-BrM  Distance (µm) | Cones | Amplitude (a.u) | EZ-to-BrM  Distance (µm) |
| 11002 | 4911 | 4.57 | 55 | 3573 | 5.52 | 54 | 964 | 4.73 | 43 | 541 | 2.47 | 38 | 307 | 2.37 | 31 |
| 11108 | 4306 | 5.21 | 50 | 2701 | 4.30 | 47 | 1427 | 3.77 | 42 | 668 | 3.04 | 34 | 350 | 2.43 | 35 |
| 11118 | 3636 | 5.98 | 50 | 3215 | 5.49 | 51 | 708 | 6.28 | 46 | 428 | 5.41 | 46 | 222 | 4.65 | 43 |
| 11121 | 4039 | 6.51 | 47 | 2955 | 4.33 | 47 | 998 | 4.16 | 39 | 428 | 2.97 | 39 | 183 | 1.82 | 31 |
| 11122 | 4035 | 3.84 | 54 | 2819 | 3.77 | 42 | 1731 | 4.17 | 38 | 480 | 2.61 | 39 | 251 | 1.46 | 35 |
| **Avg.** | **4185** | **5.22** | **51.2** | **3053** | **4.68** | **48.2** | **1166** | **4.62** | **41.6** | **509** | **3.30** | **39.2** | **263** | **2.55** | **35** |

**Supplementary Table 1.** The number of identified cones, ORG amplitudes, EZ-to-RPE/BrM distance and the corresponding averages for each participant.
